# Seventy Years of Treating Delusional Disorder with Antipsychotics: A Historical Perspective

**DOI:** 10.3390/biomedicines10123281

**Published:** 2022-12-18

**Authors:** Alexandre González-Rodríguez, José A. Monreal, Mentxu Natividad, Mary V. Seeman

**Affiliations:** 1 Department of Mental Health, Mutua Terrassa University Hospital, Fundació Docència I Recerca Mutua Terrassa, University of Barcelona (UB), CIBERSAM, 5 Dr Robert Square, 08221 Terrassa, Spain; 2 Institut de Neurociències, Universitat Autònoma de Barcelona (UAB), 08221 Terrassa, Spain; 3 Department of Psychiatry, University of Toronto, 605 260 Health Street West, Toronto, ON M5P 3L6, Canada

**Keywords:** optimizing treatment, schizophrenia spectrum, pimozide, partial D2 agonists

## Abstract

For many decades, delusional disorder (DD) has been considered a treatment-resistant disorder, with antipsychotics acknowledged as the best, though imperfect, treatment. It is possible that the discovery of the right drug could turn treatment resistance into treatment response. The goal of this narrative review is to provide a historical perspective of the treatment of DD since the introduction of antipsychotics 70 years ago. The following search terms were used to scan the literature: antipsychotics AND “delusional disorder”. Findings were that therapy for DD symptoms has changed over time. Initial reports suggested that the drug of choice was the antipsychotic pimozide, and that this drug was especially effective for the somatic subtype of DD. Subsequent studies demonstrated that other antipsychotics, for instance, risperidone and olanzapine, were also highly effective. Treatment response may vary according to the presence or absence of specific symptoms, such as cognitive defect and depression. Clozapine, partial D2 agonists, and long-acting injectable drugs may be more effective than other drugs, but the evidence is not yet in. Because of the absence of robust evidence, treatment guidelines for the optimal management of DD are not yet available.

## 1. Introduction

The first extensive description of monomania, also called paranoia, was generated by the French psychiatrist Esquirol (1772–1840). He labeled this form of mental illness a partial “délire” (French for delusion), partial because, outside of the one prominent, fiercely defended, idiosyncratic, and unchangeable false belief, the patient was generally described as fully rational [1]. The term paranoia was widely used in psychiatry until the first half of the 20th century, after which it lost its status as a stand-alone diagnosis [2]. In 1987, DSM-III-R (the revision of the third U.S. diagnostic and statistical manual of mental disorders) reintroduced the concept but gave it a new name: delusional disorder [3]. Delusional disorder (DD) is considered a serious mental disorder characterized by the presence of a fixed, preoccupying, illogical belief. It is classified as a psychotic disorder, and belongs to the schizophrenia spectrum of disorders [4]. Characteristically, delusional beliefs are based on the misinterpretation of external reality, and are not, by definition, amenable to extinction by persuasion or education [5]. Delusions in DD are sometimes accompanied by affective symptoms or perception errors; however, even when present, these do not take center stage. Whenever hallucinations do occur in DD, they are congruent with the all-consuming delusional theme [5]. The current diagnostic and statistical manuals for mental disorders classify DD into seven subtypes according to the delusional content: persecutory (belief of being persecuted or conspired against), somatic (delusional parasitosis, hypochondriasis, or body dysmorphic disorder), jealous (Othello’s syndrome), grandiose (delusions of grandeur), erotomanic (de Clérambault’s syndrome), mixed (a combination of delusional themes), and unspecified (vagueness in the expression of delusional content) [5]. These subtypes are not associated in the psychiatric literature with differential responses to available treatments.

The worldwide prevalence of DD is difficult to determine accurately because many persons with DD do not consider themselves ill, and thus, never seek treatment. The condition has been considered rare, representing only 1–4% of all psychiatric admissions. It usually never comes to medical attention until middle or late adult life, although it may begin earlier [2,6]. There are reports of DD being consistently and cross-culturally most common in low socioeconomic groups and among new immigrants. In fact, DD occurs more frequently in immigrants than schizophrenia or affective disorder [6]; except for specific local syndromes, the sociodemographic profile is consistent across different cultures [7].

The current diagnostic and statistical manual of mental disorders, fifth edition (DSM-5), defines DD as the presence of delusions that endure for at least 1 month in the absence of other psychotic symptoms [8]. While this disorder has been considered treatment-resistant, a review of cases between 1994 and 2004 [3] highlighted the fact that it is, in many instances, a treatable condition, with 50% of cases responding to antipsychotic medications. The same paper also found that depressive comorbidity was more frequent than had previously been reported. 

The most recent review on the topic investigated treatment response by including all studies that used clinician-rated scales [9]. This review found that overall response to treatment was approximately 32.3%. First-generation antipsychotics appeared to show a modest superiority over second-generation antipsychotics. This may have been an artifact of the longer duration of use of first-generation drugs at the time of publication. Very few studies controlled for treatment adherence, which is generally acknowledged as key to treatment effectiveness. 

For the third to half of cases, roughly, that do respond to antipsychotic treatment, the biological basis of treatment response remains poorly understood. Although the blockade of dopamine receptors D2 and D3 has been reported as the main mechanism of action of antipsychotics in schizophrenia, serotonergic dysfunction, and brain structure/function impairment, especially in the temporal and parietal lobes, may also be responsible for success or failure of drug therapy [10]. This is the question that prompted this review.

This paper summarizes the management of DD with antipsychotic medication from the time of introduction of the first antipsychotic, chlorpromazine. Temporally successive antipsychotic drugs are reviewed and examples of their success in treating DD are cited. 

### Aims

The goal of this review is to provide a historical perspective of the treatment of DD with antipsychotic medications. We also attempt to determine whether the mode of action of antipsychotics in DD is similar or different from that in schizophrenia.

## 2. Methods

### 2.1. Design and Data Sources

To accurately represent the literature on our topic, we conducted electronic searches through PubMed and ClinicalTrials.gov databases for English, Spanish, German, and French papers published from PubMed inception to September 2022. In addition, we searched Google Scholar to find potentially relevant papers outside of the strictly medical literature. Studies that focused on schizophrenia populations were excluded unless they contained a subsample of patients with DD.

### 2.2. Search Strategy

The following search terms were used: antipsychotics AND “delusional disorder”. Additionally, reference lists and book chapters from the initially included studies were checked in order to uncover other relevant papers in the field. 

### 2.3. Inclusion and Exclusion Criteria

Studies were only included if they met our inclusion criteria: (1) patients with delusional disorder (DD) who fulfilled the International Classification of Diseases (ICD) and the Diagnostic and Statistical Manual of Mental Disorders (DSM) criteria, irrespective of version; (2) publication in a peer-reviewed journal; (3) language restricted to English, German, French, and Spanish; (3) studies that reported the use of antipsychotic medications and referred to their putative mechanism of action, (4) studies providing a historical perspective of the treatment of DD over the last 70 years.

The following studies were excluded: (1) those of participants with DD that clearly stemmed from brain injury; (2) those where persistent delusions were clearly secondary to other psychiatric diagnoses, for instance, schizophrenia or other non-affective and affective psychoses.

### 2.4. Data Collection and Extraction

The literature search strategy, data collection, and extraction were conducted by A.G.R and M.V.S. All abstracts and titles were scanned; most were excluded because they did not address the use of antipsychotic medications in the context of DD. 

When more than one study referred to the same work, we cite the earliest publication. Based on the papers we found, we carried out a non-systematic, narrative review. The review is divided into the following sections: (1) treatment of DD before the introduction of antipsychotic medications, (2) treatment with pimozide, (3) treatment with first-generation antipsychotics, (4) treatment with second-generation antipsychotics, particularly risperidone and olanzapine, quetiapine, aripiprazole, cariprazine (5) treatment with clozapine (6) treatment with long-acting injectable antipsychotics. 

**Figure 1:**
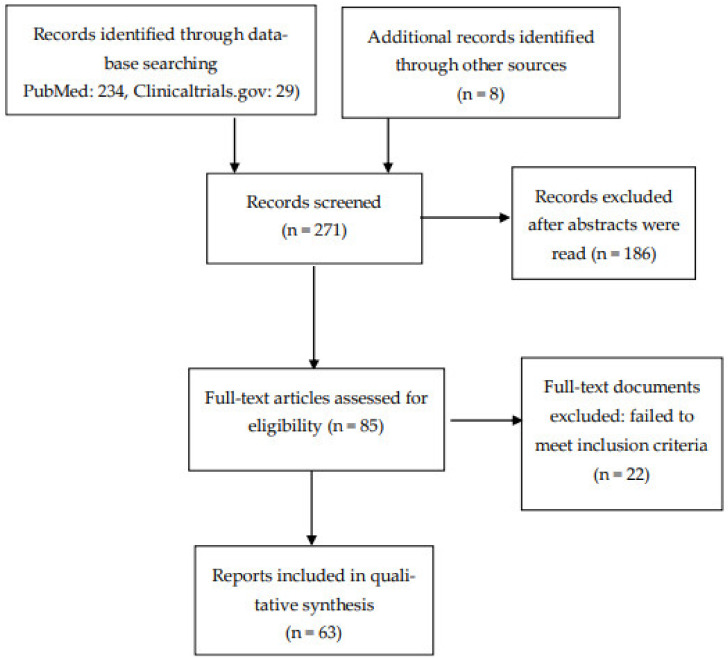
Flow diagram of included studies.

From a total of 263 records, we selected 63 articles that were relevant to our aim. Figure 1 shows the methodological procedures and results of the screening and selection process.

## 3. Treatment of Delusional Disorder Prior to the Widespread Use of Antipsychotic Medications

Before the introduction of antipsychotic medications, patients with schizophrenia and other paranoid psychoses were treated in long-term psychiatric institutions, where the emphasis was on keeping patients safe, calm, and busy. Treatment focused on safety precautions, occupational activities, and nursing care [11]. Gardening, art, music, drama, and dance therapies were some of the therapeutic modalities used in mental hospitals. Dance movement therapy was also used to improve psychological and physical well-being [12]. The mechanism by which leisure activities could improve psychotic symptoms was understudied. It was assumed that such activities were enjoyable and relaxing, encouraged in-hospital socialization, and kept patients’ minds off pathological preoccupations.

Work therapy, rest cures, and a system of rewards for appropriate behavior were also offered in psychiatric asylums. Rest cures included three main elements: rest, seclusion, and good nutrition. Massage treatment was sometimes used [13].

Malaria treatment, insulin shock, lobotomy, and electroconvulsive therapy, plus three types of hydrotherapy, were the main treatments for psychosis [14]. Hydrotherapy was described as head-out hot showers, adapted cold showers, and colonic hydrotherapy. It was hypothesized that hot and cold showers reduce stress and potentially modulate neurotransmission via the mesolimbic system of the brain.

Pharmaceutical compounds such as bromides, chloral hydrate, hyoscine, paraldehyde, barbiturates, and morphine were also used for sedation. Even after the introduction of chlorpromazine, paraldehyde was still in use as a comparator drug, specifically for toxic psychosis and delirium tremens [15,16]. Table 1 represents the main treatment options used to treat patients with psychosis, including DD, before the widespread use of chlorpromazine and related drugs.

The history of antipsychotic drug development is serendipitous, with effectiveness, until the last few decades, judged solely on the basis of clinical observations. The use of phenothiazines resulted accidentally from a search for improved antihistamines [17]. Coincidentally, Paul Ehrlich had already observed, in 1891, that methylene blue, a phenothiazine derivative, functioned as an antimalarial [18]. Chlorpromazine, developed first as an antihistamine, was noted to exert calming effects without undue sedation. It was found to calm soldiers injured on the battlefield, and to act as an analgesic during surgery. This pronounced calming effect attracted the interest of psychiatrists, who thought it might work (similar to freeze wraps) by cooling the brain. 

The discovery of the antipsychotic effect of phenothiazines in the early 1950s was part of the “psychopharmacological revolution” [19]. Chlorpromazine was shown to be effective for psychomotor agitation in acute and chronic mania, schizophrenia, and also organic psychoses secondary to lobotomy [20].

Phenothiazines and piperazines were followed by butyrophenones [20], with haloperidol being synthesized by Paul Janssen in 1958 [21]. Haloperidol replaced chlorpromazine as the most frequently prescribed antipsychotic among the many that were soon available. As more and more chemical neurotransmitters were discovered in the brain, it became generally understood that drug effects worked through inhibiting or enhancing neurotransmission, and by exerting their effects through specialized neuronal membrane receptors.

Antipsychotics were shown to be very effective in treating positive symptoms of psychosis (delusions, hallucinations, thought disorder) [22], and were used to treat these symptoms in whatever disorders they appeared.

In a long-term follow-up study that investigated the clinical course and treatment response of a cohort of 72 first-admission patients diagnosed with DD [23], clinical outcomes were compared between patients admitted during the 1946–1948 period (prior to the synthesis of chlorpromazine) and those admitted during 1958–1961. Surprisingly, it was found that the two groups fared equally poorly. In other words, antipsychotic medication did not seem to improve the outcome. The dramatic improvement seen in schizophrenia was not, at that time, apparent in DD, possibly because only the most severely ill DD patients were hospitalized.

**Figure 2:**
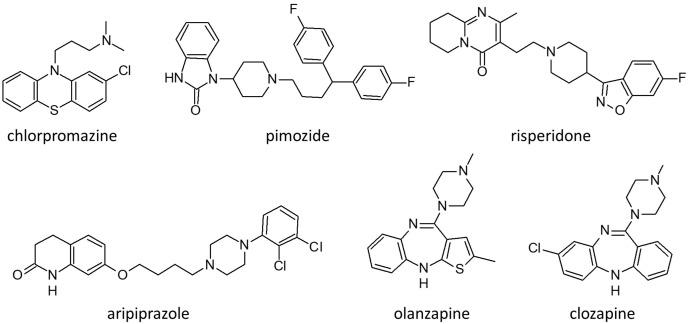
Molecular structure of the main antipsychotics used to treat patients with delusional disorders.

Table 2 summarizes the history of antipsychotic drugs in the context of delusional disorders (DDs). Figure 2 presents the molecular structure of the main antipsychotics used to treat delusional disorder: chlorpromazine, pimozide, clozapine, aripiprazole, olanzapine and risperidone.

## 4. Use of Pimozide in the Treatment of Delusional Disorder

Pimozide is an antipsychotic belonging to the diphenylbutylpiperidine class, synthesized in 1963 by pharmacologic giant, Paul Janssen. After the immense success of haloperidol, the understanding that dopamine receptor blockade was central to antipsychotic action led to the development of more butyrophenones. Serotonin antagonism subsequently emerged as contributory to the alleviation of psychotic symptoms, which led to the synthesis of pimozide, an antagonist at the D2, D3, and D4 receptors and the 5-HT7 receptor [24]. In 1975, Riding and Munro treated four cases of monosymptomatic hypochondriacal psychosis (DD somatic type) with pimozide [25]. Three of the four responded well, while the fourth showed partial improvement. Canadian psychiatrist Alistair Munro subsequently became a world leader in the treatment of delusional disorder [26], and advocated for the use of pimozide because of its specific effectiveness, especially, Munro believed, for somatic delusions such as delusional parasitosis [27]. Munro argued that, in DD, no matter the central delusional theme, the core distinction from other forms of psychosis is that the delusions are encapsulated. Though very firmly held, the delusions do not affect the entirety of a person’s life. In that sense, all themes are alike, hence there is no reason for one subtype to respond to a treatment when other subtypes do not. His explanation for the seemingly superior response of the somatic type to pimozide was that persons with somatic symptoms are more likely than others to adhere to treatment. Adherence rather than delusional content, according to Munro, was what determined response. Subsequent studies have generally agreed with this conclusion.

As well as the effects noted above, pimozide also displays actions as an antagonist, inverse agonist, and channel blocker, with relatively lower affinities, at α-adrenergic, muscarinic cholinergic, and histamine receptors and calcium and sodium channels.

Like many other antipsychotics, but more strongly than most, pimozide also inhibits the hERG (human ether-a-go-go-related) K+ channel [28]. It is the hERG block that leads to QT interval prolongation and ventricular arrhythmias, including torsades de pointes. Despite its effectiveness and advantages as an antipsychotic medication (relatively little sedation and little weight gain), it is this cardiac effect that has led to its decline in use [29]. As for its special effectiveness in delusional disorder, the Cochrane Review of 26 studies on pimozide in 2013 concluded that there were insufficient data to either support or refute this claim [24].

## 5. First-Generation Antipsychotics Other Than Pimozide

There is a large literature on first-generation antipsychotics in the treatment of schizophrenia; however, this is not the case in DD. This group of antipsychotics include chlorpromazine, haloperidol, fluphenazine, and many other extensively used drugs. They are effective dopamine 2 and 3 (D2 and D3) receptor antagonists, and work well against positive psychotic symptoms (delusions, hallucinations, and thought disorder), but show dose-related associations with extrapyramidal side effects. Such studies as exist in the context of DD suggest that the positive effect acts, as in schizophrenia, via D2 and D3 antagonism, modified perhaps because, in DD, the drugs are acting on an aging, and perhaps deteriorating, brain [10,30].

There is one report of a phenothiazine, promazine, being clinically effective in a 75-year-old woman with a 3 year history of delusional parasitosis [31]. Promazine is an aliphatic phenothiazine antipsychotic agent with low potency antidopaminergic action, α1-adrenergic antagonism, and anticholinergic properties. Its relatively low potency at dopamine receptors may be beneficial in the elderly.

Herbel and Stelmach carried out a retrospective study of 22 patients with DD receiving involuntary treatment during a 13 year period in a psychiatric hospital [32]. The vast majority of patients in this study received first-generation antipsychotics: haloperidol decanoate (*n* = 11), perphenazine (*n* = 2), fluphenazine decanoate (*n* = 3). Five patients were classified as non-responders: one received fluphenazine decanoate, one received haloperidol decanoate, and the other three received oral drugs. In theory, effectiveness depends on duration of treatment, dose, and on adherence. On the whole, duration of treatment in this study was relatively short, and doses appeared to be relatively low, which may help to explain why there were two patients that received long-acting injections, where adherence should have been guaranteed, who did not respond.

## 6. Use of Second-Generation Antipsychotics in the Treatment of Delusional Disorder

Blocking dopamine transmission at the postsynaptic receptor site is regarded as a critical action of antipsychotics. Another is the blockade of dopamine synthesis at the level of the presynaptic neuron. A longitudinal study carried out by Pomarol-Clotet and colleagues [33] examined the latter hypothesis in a group of 11 patients with DD, and 12 with schizophrenia, following a 3 month treatment period with second-generation antipsychotics. Baseline striatal dopamine synthesis was inversely associated with negative symptoms in first-episode schizophrenia, but this was not apparent in DD, which is not surprising since negative symptoms (apathy, avolition, paucity of speech, social isolation) are uncharacteristic of DD. Cheng et al. [34] explored dopamine synthesis capacity in 12 patients with schizophrenia, 11 with DD, and 12 diagnosed with other psychotic disorders. They compared results against those of 19 healthy controls. Assessment was performed using 18F-DOPA positron emission tomography (PET) and magnetic resonance imaging (MRI); psychopathological symptoms were assessed with the positive and negative syndrome scale (PANSS). The findings were that DD, schizophrenia, and related disorders all presented similar dysregulated mechanisms of dopamine synthesis, which implies that treatment with dopamine antagonists should affect all psychotic diagnostic categories to the same degree, at least if the blockade of dopamine synthesis is the main mechanism of action.

Recently, as mentioned earlier, Guàrdia et al. [10] reviewed studies of DD patients treated with second-generation drugs, and found evidence for effects on both dopamine and serotonin pathways, as well as on the mediation of brain structure impairment, particularly in temporal and parietal lobes, on drug efficacy. The implication is that older age (and thus older brains) among DD patients, compared to those with schizophrenia, may impact the response to specific therapeutic drugs and affect the dose range needed for efficacy and tolerability [3,4]. The hypothesis that serotonin pathways are important in antipsychotic action was what led to the synthesis of second-generation antipsychotics. Because they are less likely than first-generation drugs to induce extrapyramidal adverse effects, they may be especially useful for older patients.

With a particular focus on late life, Nagendra and Snowdon described consecutive cases of DD in patients referred to an old age psychiatry service [35]. Ninety-six patients received second-generation antipsychotics, and the overall response was considered positive. This suggests that second-generation drugs show superior efficacy over first-generation drugs, but these results cannot be considered as evidence. The separation between the two generations is somewhat artificial. Second-generation drugs, as a group, cause minimal extrapyramidal effects and minimal hyperprolactinemia because of greater serotonin receptor blockade and lower duration of time attached to the postsynaptic D2 receptor. Risperidone, however, classed as a second-generation drug, frequently produces extrapyramidal effects and high levels of prolactin, even at low doses. 

### 6.1. Risperidone

The vast majority of evidence on the efficacy of antipsychotic treatment of DD comes from the use of risperidone. Positive response to risperidone has been reported in patients with DD of the somatic type and other DD subtypes [36,37]. This drug is chiefly metabolized by CYP2D6. A case report by Strauss and collaborators rightly highlighted the relevance of genetic variants of CYP2D6 when treating DD with risperidone [38]. The patient was a 37-year-old woman who was a poor metabolizer of risperidone, and in whom a very low dose proved toxic. A previous study investigated the clinical response to risperidone by determining plasma concentrations of the drug, catecholamine metabolites, and CYP2D6 genotypes in a sample of 136 patients with schizophrenia, DD, and schizoaffective disorder [39]. This study found a positive association between plasma levels of risperidone plus 9-hydroxirisperidone (an active metabolite) and Simpson and Angus score (SAS), a measure of extrapyramidal effects. However, no correlations were found between antipsychotic plasma levels and PANSS scores, both of which are measures of effectiveness. Monitoring antipsychotic plasma levels may be useful when patients experience unexpectedly severe side effects from antipsychotics in general [37].

### 6.2. Olanzapine

There are recent studies on the efficacy of olanzapine in DD. Comardelle et al. [40] published the case of a 67-year-old woman with a delusion of glass in her hands and fingernails. The patient was successfully treated with olanzapine 5 mg per day in combination with psychotherapy. Freudenmann et al. and Bosmans and Verbanck [41,42] both reported on patients with DD who responded well to very low doses of olanzapine. Both patients were elderly and suffered from delusional parasitosis. As suggested by Munro, somatic discomfort may have enhanced adherence. 

Kulkarni et al. carried out a retrospective analysis of 455 patients suffering from DD [43]. The sample was divided into two groups according to the treatment received. The first group consisted of 86 patients on olanzapine, and the second of 280 patients on risperidone. Both risperidone and olanzapine were found to be effective, with no statistically significant differences between the groups in either adherence or response to treatment. Both risperidone and olanzapine are known to have side effects (extrapyramidal symptoms in the case of risperidone, and weight gain in the case of olanzapine), which could interfere with adherence. What often determines adverse effect severity is the pace of dose increase at the beginning of treatment, as well as the daily dose ultimately reached, although individual patient sensitivities to side effects are also important.

Basu et al. reported the case of a 38-year-old woman diagnosed with DD who developed restless leg syndrome (RLS) on olanzapine [44], a sleep disorder commonly associated with the use of first-generation antipsychotics. When risperidone was substituted for olanzapine, the RLS improved. This is surprising because extrapyramidal effects are usually more prevalent with risperidone than with olanzapine, which highlights the importance of individual sensitivities. By contrast, a switch from trifluoperazine to olanzapine improved tardive dyskinesia in a 59-year-old man with DD [45]; however, the olanzapine daily dose was 17.5 mg/d, which is a high dose that may have ‘covered’ tardive movements. Dose is usually more relevant to side effect severity than the choice of antipsychotic. 

### 6.3. Quetiapine

Quetiapine, another popular second-generation drug, has affinity for D2 receptors, serotonergic receptors, and alpha 1 and histaminergic receptors, but, as with most other antipsychotics, mainly works via dopamine and serotonin pathways. Prakash et al. [46] reported on a 29-year-old man suffering from DD and von Hippel–Lindau disease, a hereditary condition associated with tumors in multiple organs. The patient was treated with quetiapine 50 mg daily, which was progressively increased to 200 mg/day. Total remission of delusional symptoms was achieved. It is interesting to note that effective doses of quetiapine for schizophrenia need to be substantially higher than 200 mg/day [47], bringing forward the possibility that patients with DD, on average, do not require doses as high as those used in schizophrenia treatment.

### 6.4. Paliperidone

Paliperidone, or 9-OH-risperidone, is the main metabolite of risperidone, and comes as an extended-release (long-acting) tablet to be taken qAM. Paliperidone palmitate is also available in long-acting injectable form. Altinöz et al. [48] reported two cases of elderly patients with delusional parasitosis whose symptoms remitted after treatment with oral paliperidone; neither one experienced side effects. One patient concluded that the paliperidone had “poisoned the parasites”. The reason specific drugs work for specific patients has been attributed to individual genetics.

### 6.5. Asenapine

Asenapine is a relatively new drug that shows high affinity for serotonergic receptors, adrenoceptors, dopamine receptors, and histamine receptors. A case report by Rajkumar et al. [49] described a 44-year-old woman with a chronic and systematized delusion of jealousy. She showed a poor response to aripiprazole (a dopamine partial agonist) at 15 mg a day for 2 months. Consequently, she was changed to ziprasidone, 80–100 mg/day for 10 months. Delusions of jealousy faded, but new persecutory delusions appeared, perhaps precipitated by side effects, i.e., perioral and lingual dyskinetic movements. This combination of new symptoms and dyskinetic movements led to a diagnosis of supersensitivity psychosis, a known complication of long-term antipsychotic treatment, in which patients develop new psychotic symptoms, often accompanied by tardive dyskinesia. The syndrome, first described in 1980 by Canadian psychiatrist Guy Chouinard [50], is attributable to prolonged dopamine receptor blockade. Asenapine was chosen as the next treatment based on the belief that, because of its potent serotonin 5-HT2A receptor antagonism, it might be able to reduce D2 receptor supersensitivity. After the switch to asenapine 20 mg/day, the patient went into complete remission of psychotic symptoms, and showed no dyskinetic movements. The trouble with case histories, however, is that only successful cases are reported. Asenapine may or may not be indicated in supersensitivity psychosis. Replication is needed.

### 6.6. Ziprasidone

Ziprasidone use in DD has been reported in a few cases [49,51], most often targeted at the somatic subtype of DD. Contreras-Ferrer et al. [52] reported the case of a 73-year-old woman referred to dermatology because of deep linear ulcers on the face and arms secondary to pruritis and scratching. Delusional parasitosis was suspected. Outpatient treatment with olanzapine was not effective, thus, a combination of pimozide, ziprasidone, and dantrolene (an anticholinergic for side effects) was started in hospital. After 2 weeks on this regimen, the delusion disappeared. It may have been the separation from her family (who also believed in the parasitic infestation) that effected the cure rather than the pharmaceuticals. De Berardis et al. [53] reported the case of a 24-year-old woman who also presented with delusions of parasitosis, and was treated with ziprasidone 120 mg daily. Ziprasidone was chosen because the patient asked for a drug that would not lead to weight gain. After 1 month of treatment, the patient’s delusion began to fade, and she went into complete remission. Ziprasidone has a unique pattern of receptor affinity, and delusional parasitosis has been hypothesized to result from specific pathophysiology [54]. A good match between patient and drug could be what led to treatment success. This is contrary to the consensus that all monothematic delusions respond in the same way, but possibilities that correct matching is important to drug efficacy, such as suggested by this case history, deserve further investigation.

## 7. Use of Partial D2 Agonists

### 7.1. Aripiprazole

Aripiprazole was first patented in the U.S. in 1989 by Otsuka Pharmaceuticals, and was approved for U.S. use in 2002, in the dose range of 2–30 mg/day. It is sometimes considered a third-generation antipsychotic because of its agonist properties at presynaptic dopamine D2 autoreceptors, which prevent extrapyramidal side effects [55]. Aripiprazole’s effectiveness against psychotic symptoms, however, relies on its antagonistic function at dopamine D2/D3 postsynaptic receptors. Clinically, whether the agonist or antagonist effect is more prominent depends on dose, although individual factors must also play a role [56].

A recent systematic review explored the current evidence on the effectiveness and tolerability of aripiprazole in the treatment of DD [57]. The sample consisted of 21 patients with DD treated with aripiprazole; most were diagnosed with DD of the somatic type. All reviewed studies reported significant clinical improvement. Furthermore, aripiprazole was shown to ameliorate psychotic symptoms in some individuals who did not respond to risperidone, perhaps because extrapyramidal and hyperprolactinemic effects were avoided [58]. 

Due to its efficacy and tolerability profile, aripiprazole has been found to be effective in DD, and is particularly useful in patients with comorbid epilepsy because it lowers the seizure threshold significantly less than other antipsychotics. Garg et al. [59] reported the case of a 22-year-old man with a diagnosis of DD who suffered from systematized delusions of reference and persecution as well as generalized tonic/clonic seizures. After experiencing intolerable extrapyramidal effects from first-generation antipsychotics and suffering a major epileptic seizure during a trial of risperidone, the patient was started on aripiprazole at 5 mg/day. Sodium valproate (an antiepileptic and mood stabilizer) was added, and the aripiprazole dose was gradually increased to 20 mg per day. The patient’s symptoms improved; there were no extrapyramidal signs and no epileptic seizures. It is difficult to know whether to attribute the success to aripiprazole or to valproate; it was probably due to their combination.

Lee and Shen [60] reported the case of a 67-year-old woman initially diagnosed with dementia with psychotic symptoms. She showed aggressive behavior, poor memory, and suffered from a delusion of theft; she believed that neighbors and friends were entering her home and stealing her belongings. MRI showed mild brain atrophy with subcortical white matter small vessel ischemic changes. The patient was treated with flupentixol 0.5 mg BID, quetiapine 25 mg HS, and a rivastigmine transdermal patch (9.5 mg/24 h). After a 2 week trial, results were minimal and daytime sedation proved to be a troublesome side effect. The diagnosis was revised to delusional disorder with mild dementia and the patient was prescribed oral aripiprazole 15 mg daily along with the rivastigmine patch. This seemed to be working, but the patient refused further oral medication and was, thus, given 400 mg LAI aripiprazole/month. By the end of the month, she described only minimal delusional thinking and showed marked improvement in memory. She continued the IM aripiprazole and the rivastigmine, and at 1 year follow-up, was free of delusion. This could be attributed to aripiprazole, but suggests that the patient did not like taking pills, and, therefore, had not taken the oral medication prescribed in the past. The LAI format circumvented the problem. Another successful treatment of a 29-year-old man with delusional disorder using LAI aripiprazole was reported by Diefenderfer et al. [61]. Unfortunately, single case reports are not informative about potential mechanisms of action.

### 7.2. Cariprazine

Cariprazine blocks D3 in preference of D2 receptors; like aripiprazole, it also acts as a partial agonist at these two sites. It is also a partial agonist at the serotonin 5-HT1A receptor, and acts as an antagonist at 5-HT2B and 5-HT2A receptors [62]. Bajouco et al. reported on one case of DD treated with cariprazine [62]. The patient was a 70-year-old woman with delusions of reference and of paranoia. She had shown poor treatment adherence to various antipsychotics for the last 45 years. Despite her symptoms, she was a physician who functioned well both professionally and personally. She was free of medication between 2018 and 2020, when she suffered a relapse and was prescribed cariprazine 4.5 mg/day, which led to a remission of symptoms after 2 months of treatment. The patient remained clinically stable and tolerated the drug well at the time of the report. This might be attributable to the drug or perhaps to improved adherence.

## 8. Use of Clozapine in the Treatment of Delusional Disorder

Although clozapine is a second-generation antipsychotic, it is considered special because it is active at many different receptor sites in the brain, and thus, is effective in psychoses that are otherwise treatment-resistant. Clozapine has an interesting history. A Swiss company called Wander AG first synthesized it in 1958, based on the chemical structure of the antidepressant, imipramine. Clozapine was seen as a potential antidepressant that also showed antipsychotic properties. It seemed radically different from other antipsychotics, however, because it induced no extrapyramidal side effects. In 1987, Wander AG was bought by the pharmaceutical giant, Sandoz, which was quick to dispel the then-popular idea that neurological side effects were a prerequisite for effective antipsychotic action. Clozapine was soon shown to be effective for psychosis, but widespread use uncovered the fact that it sometimes (rarely) induced agranulocytosis, which could be fatal. This stopped its use in the U.S. The development of routine blood monitoring and the gradual recognition that antipsychotic effectiveness could be uncoupled from parkinsonian side effects brought clozapine back on the market; its special use in treatment-resistant patients was subsequently recognized [63]. 

Clozapine’s off-label use has been reported in DD. An observational Swedish registry cohort study reported on 9076 patients with DD followed up for a mean of 4.9 years [64]. The sample consisted of 4835 men and 4241 women; 2074 of the patients had required at least one hospitalization. Patients with the lowest risk for hospitalization were those treated with clozapine, after which came those treated with olanzapine or any LAI. Clozapine use was also associated with reduced risk of work disability. This is interesting, and could be explained by the regular monitoring associated with clozapine and also with LAI antipsychotics, as well as the lack of parkinsonian side effects in the case of both olanzapine and clozapine. Both close monitoring and lack of side effects are known to improve adherence to a drug regimen.

Clozapine does, however, have many serious side effects [65]. This may be a special problem for patients with DD who tend to be of middle or late age. A case report of a 53-year-old patient with DD described anticholinergic syndrome induced by a combination of clozapine with paroxetine [66]. The authors emphasized that paroxetine, as well as other selective serotonin receptor modulators, are potent inhibitors of CYP1A2, thus unduly increasing clozapine levels. Nevertheless, there is every reason to believe that patients who are willing to take drugs and who have not responded to other antipsychotics will respond if treated with closely monitored clozapine. Rodek and Kucia published the case of a 40-year-old man with no previous psychiatric history who presented with a 2 year delusion of financial poverty [67]. No improvement was achieved by outpatient treatment with escitalopram (20 mg/d), mianserin (30 mg/d), or olanzapine (5 mg/d). In hospital, escitalopram and mianserin were tapered, discontinued, and replaced by sertraline 100 mg per day, while the dose of olanzapine was increased to 10 mg per day. The dose of olanzapine was further increased to 20 mg and sertraline to 150 mg per day without improvement. The delusion paradoxically broadened to include physical health as well as poverty. The diagnosis was consequently changed from depression to delusional disorder. Olanzapine and sertraline were discontinued, and clozapine was gradually introduced up to a dose of 275 mg/day. The delusion began to fade, though it did not entirely disappear. 

## 9. Long-Acting Injectable (LAI) Antipsychotics in the Treatment of Delusional Disorder

We have already mentioned the use of LAI paliperidone and aripiprazole in DD. In general, LAI antipsychotics have been used less frequently in DD than in schizophrenia. The most recent study was an observational Swedish registry study carried out by Lähteenvuo et al. [64], who found that, in general, long-acting injectable antipsychotics reduce the risks of hospitalization and work disability in DD. This advantage is more important in this condition than in schizophrenia because most patients with schizophrenia are unemployed, whereas many patients with DD hold jobs. 

González-Rodríguez et al. [68] investigated the effectiveness of long-acting injectable antipsychotics in the treatment of DD patients, some of whom also reported hallucinations [68]. A total of 45 outpatients were followed for 6 months; psychopathological symptoms were evaluated by means of the PANSS, and depressive symptoms by the 17-item Hamilton rating scale for depression (HRSD-17). After adjustment for potential confounding factors, patients receiving long-acting injectable antipsychotics showed lower scores in the PANSS negative subscale, and a tendency toward more improvement in positive psychotic symptoms, such as hallucinations. The same team carried out a longitudinal retrospective study with a 1 year follow-up of DD patients admitted to a psychiatry ward over a 10 year period [69]. In total, 23 patients received risperidone long-acting injection (RILD), 30 oral risperidone, and 25 were given oral second-generation antipsychotics. The group receiving RILD were more likely to stay in treatment and to require lower doses of adjuvant antidepressants and benzodiazepines than the other groups. The important advantage of LAI antipsychotics is that they improve adherence. When patients miss appointments for injections, the treatment gap is noticed by staff, who then reach out to reluctant patients. 

The timeline of the availability of long-acting injectable antipsychotics is presented in Table 3. LAI antipsychotic drug development has been classified into three groups: first-generation antipsychotics, second-generation antipsychotics, and partial D2 agonists.

## 10. Discussion

Since the introduction of antipsychotic medications 70 years ago, there has been a continuing search for the right drug for the right psychotic syndrome. 

The main aim of this review was to provide a historical perspective of the treatment of DD with antipsychotic medications, and to determine whether the mechanism of action of antipsychotics in DD is similar or different from that in schizophrenia. 

A study comparing clinical outcomes in 72 first-admission patients with DD did not find any differences in clinical outcomes between groups admitted before and after the introduction of antipsychotics [23]. This implied that antipsychotics were not effective in DD, but subsequent reports demonstrate that this is not the case. 

For a time, the drug pimozide, which is an antagonist at the D2, D3, D4, and 5-HT7 receptors, was thought to be specific for DD, but this too, has been proven wrong [24,25,26,27]. Pimozide is equally effective in other psychotic syndromes, but is currently rarely used because of cardiac toxicity. Similarly, all antipsychotic medications are effective in DD treatment, though tolerability and, thus, adherence differ [36,37,38,39,40,41,42,43,44,45,46,47,48,49,50,51,52,53,54,55,56]. The mechanism of action is generally attributable to action on dopamine and serotonin pathways, though other neurotransmitters may also be involved. In the particular case of schizophrenia, a specific glutamate receptor, the N-methyl-D-aspartate (NMDA) receptor, has been implicated in the pathophysiology of schizophrenia [70]. Glutamate and GABA abnormal concentrations have been found in the prefrontal cortex in schizophrenia, suggesting that neurotransmitter systems, other than dopaminergic systems may be associated with treatment response in psychosis [71].

Many case studies show that one particular drug, often clozapine or a long-acting injectable antipsychotic, can be superior to previous regimens, but this probably relates to side effects and adherence, not to intrinsic efficacy. The study by Lähteenvuo and collaborators, the largest cohort study to date [64], bears this out.

The use of partial D2 agonists has advantages in terms of side effects [62]. Furthermore, other psychotropic medications, such as antidepressants, are often needed. The addition of non-pharmacological treatments such as cognitive behavioral therapy may be beneficial [72]. Combining a variety of treatments, as is conducted in schizophrenia, may help to overcome treatment resistance.

It has not been shown, but it is possible that, in the future, specific drugs, with specific modes of action, will be targeted at specific symptoms.

## 11. Conclusions

The aim of this review was to provide a historical perspective of the treatment of delusional disorder with antipsychotic medications. An important finding is that the literature on mechanisms of action of antipsychotics, specifically in DD, is very sparse. It is assumed that the mechanisms are identical to those in schizophrenia, but this may not be the case because DD patients are generally older, and aging brains may well respond differently to this class of drugs. However, we found no evidence that the response differs among drugs, although individual persons, of course, show individual responses. It may be that specific psychotic symptoms respond best to specific drugs, but this has not been demonstrated. It may be that specific domains of delusional thinking (strength of conviction, intensity of preoccupation, sensory perception, misidentification, reasoning capacity) may respond differentially to treatment, but this has yet to be investigated. It may also be that DD patients require a combination of pharmacologic and psychosocial treatments rather than drugs alone. Because of the absence of robust evidence, 70 years after the introduction of antipsychotics, treatment guidelines for the optimal management of DD are not yet available.

## Figures and Tables

**Table 1:** Treatment of delusional disorder before the introduction of antipsychotics.

Treatment for Psychosis Prior to Chlorpromazine		
Asylum Care	Procedures	Pharmaceuticals
Gardening	Malaria treatment	bromides
Art/Music	Freeze wraps	chloral hydrate
Dance/Theatre	Hydrotherapy	hyoscine
Rest cures	Insulin shock	paraldehyde
Token economy	Lobotomy	barbiturates
Work therapy	Electroconvulsive therapy	morphine

**Table 2:** Development of first- and second-generation antipsychotic medications.

First-Generation Antipsychotics			Second-Generation Antipsychotics, Including Partial D2 Agonists			
1952	1960s	1970s	1980s	1990s	2000s	2010s
CPZ	Halo Perph Fluph Thio Loxap Triflu	Pimoz	Cloz	Risp Olan Quet Zipr	Aripip Palip Iloper	Asena Luras Caripr

Abbreviations: CPZ–chlorpromazine; Halo–haloperidol; Perph–perphenazine; Fluph–fluphenazine; Thio–thioridazine; Loxap–loxapine; Cloz–clozapine; Risp–risperidone; Olan–olanzapine; Quet–quetiapine; Zipr–ziprasidone; Aripip–aripiprazone; Luras–lurasidone; Caripr-cariprazine.

**Table 3:** History of the development of long-acting injectable (LAI) antipsychotics.

First-Generation Long-Acting Injectable Antipsychotics			Second-Generation and Partial D2 Agonists Long-Acting Injectable Antipsychotics			
1970s	1980s	2003	2010–2020s			
LAI: fluph decanoate	LAI: halo decanoate	LAI: risp microspheres	LAI: palip palmitate	LAI: olanz pamoate	LAI ari	LAI: palip 3 and 6 monthly

Abbreviations: Ari, Aripiprazole; D2, Dopamine 2; Fluph, Fluphenazine; Halo, Haloperidol; LAI, Long-Acting Injectable; Olanz, Olanzapine; Palip, Paliperidone; Risp, Risperidone.

## Data Availability

The data presented in this review are available on request from the corresponding author.
